# Bacteria That Cause Enteric Diseases Stimulate Distinct Humoral Immune Responses

**DOI:** 10.3389/fimmu.2020.565648

**Published:** 2020-09-16

**Authors:** Souwelimatou Amadou Amani, Mark L. Lang

**Affiliations:** Department of Microbiology and Immunology, University of Oklahoma Health Sciences Center, Oklahoma City, OK, United States

**Keywords:** humoral immune response, plasma cell, memory B cell, antibodies, enteric bacteria

## Abstract

Bacterial enteric pathogens individually and collectively represent a serious global health burden. Humoral immune responses following natural or experimentally-induced infections are broadly appreciated to contribute to pathogen clearance and prevention of disease recurrence. Herein, we have compared observations on humoral immune mechanisms following infection with *Citrobacter rodentium*, the model for enteropathogenic *Escherichia coli, Vibrio cholerae, Shigella* species*, Salmonella enterica* species, and *Clostridioides difficile*. A comparison of what is known about the humoral immune responses to these pathogens reveals considerable variance in specific features of humoral immunity including establishment of high affinity, IgG class-switched memory B cell and long-lived plasma cell compartments. This article suggests that such variance could be contributory to persistent and recurrent disease.

## Introduction

Enteric pathogens rapidly activate host innate and adaptive defense mechanisms upon infection. These mechanisms include activation of innate immune cells, their production of cytokines and chemokines, and antigen presentation necessary for the recruitment of inflammatory cells (1), and initiation of the adaptive immune response (2).

**Figure 1:**
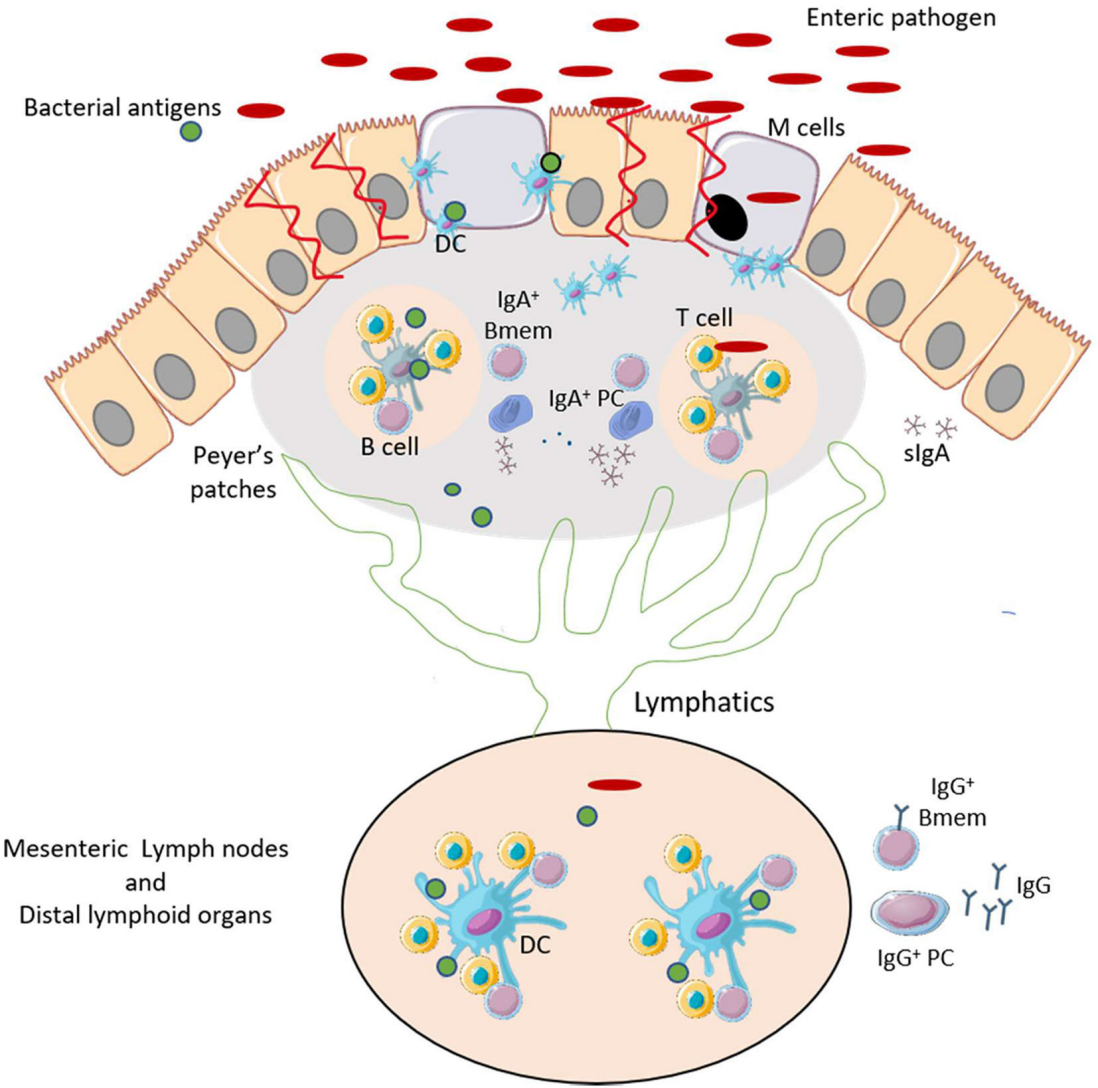
Enteric pathogens promote an inflammatory response and can induce gut-associated mucosal humoral immunity. After infection by enteric pathogens, bacteria and bacterial antigens may be uptaken by M cells. This causes an inflammatory response that promotes recruitment and maturation of DCs. The primed DCs activate T-helper cells in the lymphoid follicles of the Peyer's Patches. T cells interact with B cells to induce development of IgA^+^ memory B cells (Bmem) and plasma cells (PCs), and production of antigen-specific IgA. The bacterial antigens may reach the mesenteric and distal lymphoid organs via the lymphatic system. This leads to the production IgG antibodies encoded by Bmem cells and PCs. This figure was prepared by modifying Servier Medical Art, licensed under a Creative Common Attribution 3.0 Generic License. http://smart.servier.com/.

**Figure 2:**
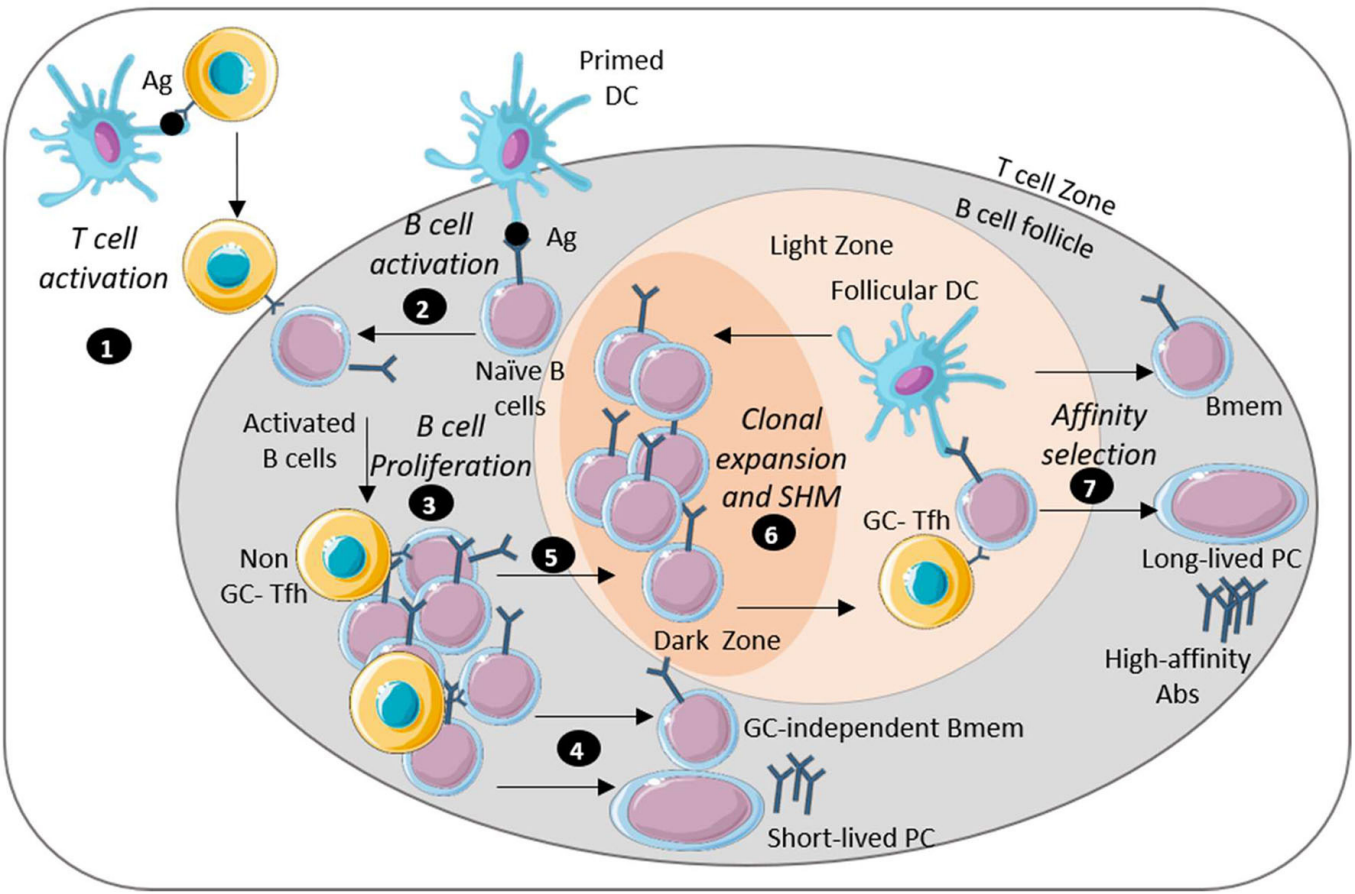
Long-lasting humoral immunity is mediated by long-lived plasma cells and memory B cells. Image depicts key steps in the generation of sustained humoral immunity: (1) APC-activated antigen-specific T cells migrate near the B cell follicle. (2) Naïve B cells encounter antigen through interaction with DCs and move by the T cell border. (3) B cells interact with the T cells differentiate into Tfh cells and migrate into the B cell follicle where activated B cells undergo proliferation. (4) Some extra-follicular B cells differentiate into antibody-secreting short lived plasma cells or GC-independent memory B cells. (5) Some B cells also enter the germinal center (GC). (6) The reaction in the GC starts with a rapid proliferation of the B cell leading to isotype-switch and BCR affinity maturation. (7) The B cells then undergo survival selection based on their affinity for antigen. Some post-GC B cells emerge as memory B cells or long-lived plasma cells that encode high affinity antibodies. This figure was prepared by modifying Servier Medical Art, licensed under a Creative Common Attribution 3.0 Generic License. http://smart.servier.com/.

Bacteria such as *Vibrio cholerae* and *Clostridioides difficile* secrete enterotoxins that mediate the pathogenesis and the inflammatory responses which often leads to tissue injury and loss of intestinal barrier integrity. Other bacteria such as *Salmonella* and *Shigella* overcome the intestinal barrier through invasion via the microfold cells (M cells) which are specialized epithelial cells that overlie Peyer's Patches of the intestine (3–5). Transcytosis of the bacteria by M cells facilitate their colonization of the gut mucosa and promote the induction immune responses in the Peyer's patches (6) (Figure 1). Depending of the efficiency of the immune response and the pathogenicity of the bacteria, the infection can be cleared after an inflammatory response and with limited intestinal tissue damage. A localized inflammatory response may include recruitment and activation of dendritic cells (DCs). The activated DCs migrate within the Peyer's patches and to the draining mesenteric lymph nodes to initiate T and B cell responses. These responses lead to the production of significant amounts of mucosal IgA and some systemic IgG that can traffic back to the gut (7, 8). However, with certain bacteria, a loss of intestinal barrier integrity may be needed to facilitate bacterial dissemination through the bloodstream resulting in a systemic infection. The damage may allow the secreted virulence factors and other bacterial antigens to reach distal lymphoid organs via the lymphatic system (Figure 1). These actions may result in systemic B cell and T cell responses. This extra-intestinal adaptive response summarized in Figure 2, is necessary for the production of high affinity, antigen-specific IgG antibodies in the germinal center (GC) (9).

In this article, we present an overview of what is known about the host immune responses to five enteric pathogens: *C. rodentium, V. cholerae, Shigella* spp., *Salmonella* spp., and *C. Difficile*, with an emphasis on humoral immunity and B cell memory. We discuss the humoral immune responses to those pathogens and the extent to which infection may induce a protective response, as well as gaps in our understanding of these processes. This information may be beneficial for understanding the course of disease.

## Citrobacter rodentium

*Citrobacter rodentium* is a murine-specific Gram-negative extracellular bacterial pathogen that is related to the human enteropathogenic and enterohemorrhagic *Escherichia coli* (EPEC and EHEC, respectively). *C. rodentium* infection is a well characterized murine model of infectious colitis (10, 11). The disease induced by *C. rodentium* is dependent on the strain and the age of mice. In most strains, including Swiss Webster and C57BL/6, adult mice develop a mild self-limiting enteric disease. However, younger mice and strains such as C3H/HeOu, FVB, and C3H/HeN have a more severe disease characterized by diarrhea and severe colitis associated with weight loss, rectal prolapse, and death due to dehydration (12–14). Colonization and disease severity are also dependent on the intestinal microbial population. The variation in production of short-chain fatty acids (SCFA) determines the susceptibility to infection. A microbiome rich in butyrate-producing bacteria impairs growth of *C. rodentium* and protect against infection and disease (15, 16).

*C. rodentium* is transmitted through the fecal-oral route. After inoculation of mice, the bacteria first establish themselves in the caecal lymphoid patch and disrupt the commensal flora. Then the bacteria expand throughout the gastrointestinal tract to colonize the distal colon and the rectum (17–19). *C. rodentium* colonizes the gastrointestinal tract of mice via the mechanism of attaching and effacing (A/E) similarly to EPEC and EHEC (20). The pathogens adhere and bind intimately to epithelial cells through the adhesin intimin and induce pedestal-like protrusions at the site of attachment. The type III secretion system (TTSS) of the bacteria injects several virulence effectors into the host cells to interfere with signal transduction, modify actin cytoskeleton, and inhibit microtubule function. A/E bacteria also secrete virulence factors that promote junctional disruption of the epithelial cells and loss of intestinal barrier (21). These actions lead to damage of the brush border microvilli, intestinal inflammation and formation of plaques of cytoskeletal filaments underneath the adhering bacteria (19, 20).

Upon infection, *C. rodentium* activates host innate receptor TLR4 which then promotes recruitment of inflammatory cells including neutrophils. Neutrophil infiltration in the infected gut significantly contributes to the formation of crypt abscesses. TLR4-deficient mice have decreased bacterial colonization and tissue damage (22, 23). In contrast, infection with the EPEC and EHEC in humans is associated with a weaker inflammatory response despite the ability of the bacteria to cause disruption of epithelial barrier integrity. This can be explained by the bacteria ability to inhibit various MAP kinase pathways associated with NF-κB and induction of innate immune responses (24, 25). Although the initial host response to the infection contributes to *C. rodentium* pathology, this immune response also plays a protective role. The myeloid differentiation primary response protein (MyD88) is necessary for limiting bacterial colonization and promoting clearance. MyD88-dependent Toll-like receptor 2 and 4 (TLR2 and TLR4) signaling mediates production of pro-inflammatory cytokines such as Tumor necrosis factors (TNF), and recruitment of neutrophils, macrophage and innate lymphoid cells (ILCs) dependent on Interleukin 6 and 23 (IL-6 and IL-23) (26–28). At the early stage of the infection, Type 3 ILCs (ILC3) produce IL-22 which promotes maintenance of the epithelial barrier and control of bacterial burden by inducing production of antimicrobial peptides (29–31). This rapid production of cytokines and chemokines, and the recruitment of leukocytes required for the induction of a protective immune response is mediated through the activation of the intestinal G-protein couple receptors by the SCFAs.

The protective adaptive immune response against *C. rodentium* is mediated by CD4^+^ T cell and B cell responses (32, 33). Infection with *C. rodentium* induces a strong mucosal IgA response but also systemic IgM and IgG responses specific to several antigens such as the adhesin intimin, a TTSS effector protein (34, 35). However while serum IgG antibodies are required for protection against disease and bacterial clearance, IgA and IgM are dispensable (36). Patients with EHEC have also been shown to mount a strong IgA, IgG and IgM responses to *E. coli* O157:H7 intimin (37).

The neonatal Fc Receptor (FcRn) has been shown to mediate transport of IgG from the blood to the intestinal lumen to mediate defense against *C. rodentium*. Infection induced IgG antibodies, mostly IgG1, mediates control of the bacteria by activating complement and inducing engulfment of the bacteria by neutrophils (38). The FcRn is also involved in the transport of antigen out of the lumen to the local lymphoid organs where it initiates a systemic immune response (39–41). This protective antibody-mediated immune response against *C. rodentium* is predominantly regulated by T follicular helper (Tfh) cells. Following *C. rodentium* infection, Tfh cells expand in the mesenteric lymph nodes and spleen, and secrete IL-21 and IL-4 (41). Infection also promotes rapid germinal center responses (42). Other CD4^+^ T cells such as Th1 and Th17 cells are also involved in the host response against the bacteria through production of IL-17, IL-22, and IFN-γ. T cell activation promotes protective intestinal IgA and serum IgG responses (41, 43–46). The activation and recruitment of effector T cells also requires production of SCFAs. These metabolites promote intestinal antibody responses in mice infected with *C. rodentium* (47, 48). CD4^+^ T cells are also involved in the cellular response to EHEC in cattle. Following experimental colonization of cattle with EHEC 0157:H7, bacteria-specific T cells infiltrate the calves' rectal mucosa, secrete IFN-γ and demonstrate antigen-specific proliferation to TTSS effectors (49).

## Vibrio cholerae

Cholera is an acute, severe diarrheal disease that remains an important global public health problem with up to 4 million cases and 143,000 associated deaths annually. The disease is caused by *Vibrio cholerae*, a highly motile Gram-negative facultative bacteria (50, 51).

*V. cholerae* is transmitted via the fecal-oral route following ingestion of contaminated water or food. After ingestion, most bacteria die because of the acidic environment of the stomach. The surviving bacteria adhere to and colonize the small intestine. The diarrhea is due the secretion of a very potent enterotoxin (cholera toxin- CT) which consists of two different subunits (A and B). Cholera toxin B binds to intestinal brush border cells leading to the endocytosis of subunit A which targets adenylyl cyclase and increases cyclic adenosine monophosphate (cAMP) levels. The increase in cAMP causes excessive secretion of chloride ions which results in the accumulation of water in the gut (52). Beside the toxin, the toxin co-regulated pilus (TCP) is also an important virulence factor. TCP is required for bacteria colonization. It provides a matrix that allows the bacteria to aggregate protecting them from host immune response (53).

*V. cholerae* is a non-invasive pathogen and doesn't induce a strong inflammatory response. Nonetheless, the bacteria penetrate the mucosa of the small intestine and attach to its surface to the small intestinal epithelial cells. Colonization of the intestine leads to structural changes to the epithelium including the widening of the intracellular spaces and alterations of the apical junctions (54). Those changes trigger rapid infiltration of inflammatory cells mainly neutrophils, macrophages, and dendritic cells. *V. cholerae* upregulates mucosal innate defense factors including TLR8, NLRP3 inflammasomes, NF-κB and MAPK signaling pathways (55–59). However, cholera toxin dampens innate immune response by inhibiting macrophage production of pro-inflammatory effectors such TNF, nitric oxide (NO) and IL-12 and by increasing secretion of the anti-inflammatory cytokine IL-10 (60). Also, *V. cholerae* has developed resistance against antimicrobial peptides by reducing their levels using its outer membrane vesicles (OMVs) and efflux pumps, and by inhibiting their binding (61–63).

Natural *V. cholerae infection* induces a protective adaptive immune response that is initiated by the activation of both B and T cells in the Peyer's patches of the intestinal mucosa and their subsequent migration into the mesenteric lymph nodes (64). Shortly after infection, bacterial antigen-specific lymphocytes are detected in the circulation. The lymphocytes express gut homing chemokine-receptors that allow homing in the intestinal mucosa where they lead mucosal immune responses. Infection induces mainly an IL-13 secreting Th2-mediated response, and some IFN-γ - secreting CD4^+^ and CD8^+^ T cell responses (65). *V. cholerae* infection also induces the development of circulating Tfh cells. These Tfh cells provide help to activated B cell leading to secretion class-switched antibodies by plasma cells and development of antigen-specific memory B cells (66).

The infection induced plasma cell response is characterized by the development of both systemic and gut derived *V. cholerae-*specific antibody secreting cells (67–69). Consequently, patients have high levels of anti-cholera toxin as well as anti-LPS IgG and sIgA antibodies. Anti-LPS sIgA in the fecal samples of patients correlates with protection against disease. However, the plasma cell response is short-lived and both systemic and mucosal antibodies significantly decrease within a few months post infection (70, 71). Natural infection by *V. cholerae* also *induces* memory B cell responses specific to various antigens including the cholera toxin, the subunit A of TCP, LPS, and the O-specific polysaccharides (OSP). These memory B cells induce a robust and rapid recall response upon reinfection and play a significant role in a longer-lasting protection against cholera (72–74). This long-lasting immune response is mediated specifically by LPS and OSP-specific IgG memory B cells. Anti-toxin IgG or anti-toxin and anti-LPS IgA memory B cells do not correlate with the long-term immunity against *V. cholerae* (75, 76).

The microbiome also plays an important role in protection and recovery against the infection. In children with cholera, clinical recovery correlate with a diverse and rich microbial community and a high concentration of SCFAs (77). SCFAs included acetate, propionate and butyrate inhibit the action of CT on the colon and prevent loss of fluid and electrolytes (78). The metabolites also facilitate anti-CT antibody responses by promoting dendritic cell functions and expression of plasma cell differentiation genes (48).

## Salmonella enterica

*Salmonellae* are Gram-negative, flagellated, facultative anaerobic bacteria that cause disease in various hosts. The clinical disease in humans is typically caused by different serovars of *Salmonella enterica* subspecies (79). Typhoidal *Salmonella* serovars, including Typhi, Sendai, and Paratyphi A, B, or C, exclusively infect humans and cause a life-threatening enteric fever. Non-typhoidal *Salmonella* (NTS) serovars, such as *S*. Typhimurium, *S* Enteritidis, and *S*. Dublin cause a gastroenteritis which can manifest with acute diarrhea, abdominal pain, fever and vomiting. In some cases, largely in infants, older individuals and the immunocompromised, NTS can cause an invasive infection including bacteremia meningitis and septic arthritis (80).

*Salmonellae* are transmitted via the fecal-oral route through contaminated food or water, person-to-person contact, or contact with animals (81, 82). After ingestion, the bacteria survive the acidic environment of the stomach using a pH homeostasis mechanism. This mechanism is triggered by the low pH of the stomach. It allows the bacteria to maintain an internal pH above 5 and prevent severe acid stress (83). Then, the bacteria migrate into the small intestine and invade intestinal epithelial cells. *Salmonellae* preferably adhere to and translocate into the Microfold cells (M cells) of the small intestinal epithelium that overlie the Peyer's patches (4, 84). Invasion of the intestinal epithelial cells is mediated by the *Salmonella* Pathogenicity Island (SPI)-encoded virulence factors including two distinct TTSSs. The TTSSs transport their effectors in the host cells where they disrupt the actin and microtubule cytoskeletons and the cell membrane to form a membrane ruffle that facilitates engulfment of the bacteria. In the cytoplasm, the bacteria replicate in a vacuole, termed the *Salmonella* containing vacuole (SCV), that transcytoses to the basolateral membrane and allows invasion of the submucosa (85–88). The bacteria cells are subsequently phagocytosed by macrophages in the intestinal submucosa where they replicate within the SCVs (89). Migration of the infected macrophages facilitates dissemination of the bacteria, which in some cases allows for the establishment of a systemic infection (90). The infection to the pathogens is also modulated by the microbial community of the gut. A microbiome rich in SCFAs-producing bacteria inhibit motility, biofilm formation and gene expression of *S. enterica* (91).

The early host response to infection by NTS is triggered by the detection of TLRs. Activation of TLRs leads to production of proinflammatory cytokines such as TNF, IL-1β, and IL-6 which promote the production of anti-microbial peptides and the recruitment of neutrophils and macrophages in the mucosa (92–94). During NTS infection, neutrophils play an important role in the early host defense but also in the development of gastroenteritis. While neutrophils prevent bacterial replication and limit their dissemination (95), they also contribute to intestinal tissue damage and loss of barrier integrity, leading to increased inflammation and diarrhea (96).

The adaptive immune response to infection by NTS such *S*. Typhimirium starts with an early DC-mediated activation of pathogen-specific CD4 T cells limited to the Peyer's patches and the mesenteric lymph nodes (97, 98). This CD4 T cell response is mainly mediated by Tbet-expressing, IFNγ-secreting Th1 cells, and it is necessary for resistance to the infection and final clearance of the bacteria in the tissues (99, 100). However, in the early stage of the infection and in the absence of CD4 T cells, CD8 T cells and NK cells secrete IFNγ and are able to control the bacterial load (101, 102). *Salmonella enterica* serovars including Typhimirium have evolved mechanisms to limit DC function and evade T cell immunity. This evasion mechanism is mediated by *Salmonella* TTSS and effector proteins (103). Passive immunization against NTS has been shown to be protective, and IgA is not essential for this protection. Also, it has been shown that B cells are required for resistance against secondary infection. However, the mechanism of protection is independent of secreted antibodies (104–107).

During the course of infection, *S. typhi* induces an early extra-follicular, low affinity antibody response, consisting largely of non-class-switched IgM. The germinal center reaction and the production of high affinity antibodies are significantly delayed but this does not prevent clearance of the bacteria (108). Both typhoidal and NTS have been shown to impair plasma cell responses. An *S. typhi* adhesin protein, SiiE has been shown to reduce IgG-secreting cells in the bone marrow suggesting that the plasma cell response following *Salmonella* infection may not be long-lived. Also *S*. Typhi infection may abrogate established long-lived plasma cell response against previous infections (109). Very little is known about the *Salmonella* specific memory B cell response and the role it plays during infection. However, it has been shown that the outer membrane proteins of *S*. Typhi induces the development of IFNγ-secreting T follicular cells and IgM^+^ memory B cells (110).

## Shigella

*Shigella* species are Gram-negative, non-spore forming, facultative anaerobic bacteria, including *Shigella sonnei, S. boydii, S. flexneri*, and *S. dysenteriae* that cause shigellosis. Shigellosis is an acute mucosal inflammation that leads to symptoms ranging from abdominal pain and mild diarrhea to severe dysentery, and sepsis (111). There are about 125 million cases of *Shigella*-associated diarrhea each year, resulting in more than 160 thousand deaths worldwide particularly in young children (112).

*Shigella* is transmitted via the fecal-oral route directly from one person to another or through contaminated water or food, and is highly infectious (113). After ingestion, the bacteria survive the acidic environment of the stomach and migrate to the colon and the rectum (114). The bacteria are non-motile and do not adhere to the colon but are able to invade and colonize the colonic epithelia (5). The bacteria break the intestinal epithelial barrier by invading the M cells that overlie the Peyer's patches (115, 116). They are translocated by M cells and are phagocytosed by macrophages. The bacteria escape the phagosome and quickly induce macrophage cell death by apoptosis. The bacteria are released and they propagate in the intestinal submucosa (117–119). Then, they enter the basolateral side of epithelial cells by micropinocytosis (120). The *Shigella* Type III secretion system (TTSS) secretes various effectors encoded in the *Shigella* virulence plasmid (121). Along with the TTSS, the invasion plasmid antigens (IpaB, C, and D) are the major virulence factors of *Shigella*. They are essential for bacterial invasion and intracellular survival, mediate the secretions of other effectors, and are dominant immunogenic antigens (122–124). Other TTSS effectors mediate polymerization of actin filaments and reorganization of the cytoskeleton by activating small GTPases. The restructuring of the cytoskeleton facilitates uptake of bacteria by epithelial cells and invasion of host cells (125–128). Some *Shigella* strains secrete toxins including the cytotoxic Stx1 and Stx2, and two other enterotoxins that may play a role in the early phases of *Shigella*-associated diarrhea (129–131).

The initial host immune response against *Shigella* is characterized by a severe and acute inflammation mediated by Caspase-1 activation of IL-1β and IL-18 following infection of macrophages and epithelial cells (117, 132). Invasion of *Shigella* can also lead to IL-8 secretion triggering massive neutrophil infiltration (133). The effect of IL-8 limits bacterial translocation but also worsens neutrophil-mediated intestinal epithelial damage (134). Besides inducing macrophage death, *Shigella* utilizes various mechanisms to counteract the host innate immune response. Through the TTSS and its effectors, *Shigella* dampens inflammatory responses to facilitate bacterial colonization by inhibiting ATP-dependent endogenous danger signaling, manipulating NF-κB pathways, and regulating expression of some pro-inflammatory cytokines and anti-microbial peptides (135–138). The administration of SCFAs may counteract this action in patients with shigellosis as the SCFAs modulate inflammation and increase the production of the anti-microbial peptides LL-37 (139). IFN-γ is crucial for the control of bacterial colonization and recovery from acute infection, however studies suggest that its levels are downregulated during shigellosis (140, 141).

Adaptive immunity, including antibody-mediated responses, have also been shown to play an important role in protection against *Shigella* infection. Following infection, the antibody-mediated immune responses are characterized by mucosal and systemic responses specific to *Shigella* lipopolysaccharide (LPS) antigens, the invasion plasmid antigens (Ipa), shiga toxins, and other TTSS effectors. These responses are *Shigella* serotype-specific and there is no cross-protection against infection from different strains (142, 143). Systemic *Shigella* LPS-specific IgG antibodies appear to be a good correlate of protection against shigellosis (144). Natural infection elicits the development of *Shigella*-specific IgA plasma cells detectable in the peripheral blood of patients. Contrary to immunization with live attenuated *Shigella* which elicits protective antigen-specific IgG and IgA Bmem cells, initial challenge with *Shigella* does not appear to induce development of antigen-specific IgA or IgG Bmem cells. However, there is an increase in the frequency of circulating LPS-specific IgA Bmem cells following a secondary *Shigella* challenge that negatively correlates with disease severity (145–148). Also, *Shigella* outer membrane protein A has been shown to enhance the germinal center reaction and antibody affinity maturation (149). Moreover, *Shigella* infection induces both primary and recall T cell expansion, predominantly of Th17 cells. Th1 cells are induced at a low frequency following reinfection, and *Shigella*-specific Th2 and CD8^+^ T cells are not detectable (150). T cells are essential for clearance of bacteria during primary infection, and *Shigella*-specific IL-17A secreting T cells are crucial for limiting bacterial growth during reinfection (150).

There is evidence of natural *Shigella* infections inducing serotype specific adaptive immune responses against recurrent infection. However, protection appears to only occur after multiple episodes, and the responses are slow and short-lived especially in young patients who have had less exposure to the infection (146, 151, 152). This can be attributed to the fact that *Shigella* manipulates the host adaptive immune response by targeting DCs, T and B cells. *Shigella* downregulates DC recruitment during infection by decreasing production of the chemokines and cytokines such as CCL20 (138). *Shigella* effectors also mediate apoptotic death of DCs (153). Moreover, studies have shown that *Shigella* invades T cells and impacts their function and dynamics in the lymph nodes by inhibiting chemokine-mediated migration (154, 155). Lastly, *Shigella* suppresses or evades the adaptive immune response by inducing B cell apoptosis both *in vitro* and *in vivo* during shigellosis through interaction between the TTSS effector IpaD and TLR2 (156).

## Clostridioides difficile

*Clostridioides difficile* is a Gram-positive, spore-forming, obligate anaerobe and the main causative agent of hospital-acquired diarrhea (157, 158). The Centers for Disease Control and Prevention (CDC) has classified *C. difficile* as an urgent threat. The CDC reported that in 2017, there was an estimated 223,900 cases of *C. difficile* infection (CDI) in hospitalized patients resulting in 12,800 deaths and costing the healthcare system more than one billion dollars (159). Although healthcare-associated cases have been declining, there is a growing number of community-acquired cases which represent 41% of CDIs (160). The *C. difficile*-associated gastrointestinal disease (CDAD) ranges from mild diarrhea to pseudomembranous colitis, toxic megacolon, or sepsis. CDI is further complicated by a high and increasing frequency of recurrence; up to 35% of patients will relapse with often more severe disease (161).

Intestinal homeostasis, the diversity in the microbial community and the abundance of health-associated metabolites constitute an effective mechanism of resistance to *C. difficile* colonization. The disruption of the microbiome often due to prolonged use of broad-spectrum antibiotics is a major risk factor for infection. CDI is characterized by the depletion of essential bacteria that produce SCFAs and butyrate which normally alleviate the pathogenicity of infection (162, 163). *C. difficile* is transmitted via the fecal-oral route. After ingestion and upon loss of colonization resistance factors, the spores resist the acidity of the stomach, germinate upon exposure to bile acids in the small intestine and adhere and colonize the large intestine (164). CDAD is mainly caused by the two secreted toxins, toxin A (TcdA) and toxin B (TcdB). The toxins target the colonic epithelium by inducing cytoskeleton condensation leading to cell death and loss of intestinal barrier integrity (165). Besides TcdA and TcdB, *C. difficile* can produce other virulence factors that contribute to motility, adherence and colonization including binary toxin (CDT), flagella, adhesins such the surface layer proteins (SLPs), and hydrolytic enzymes (166).

The host innate immune response is thought to play both pathogenic and protective roles during CDI. Toxins induce the production of pro-inflammatory cytokines and other immune mediators such as IFN-γ, IL-1β, TNF, and leptin that may contribute to inflammation and intestinal damage (167–171). Conversely, the toxins and other virulence factors including the flagella and SLPs activate NOD-1 and TLR5. This in turn promotes recruitment of neutrophils which play an important role in early defense against CDAD. *C. difficile* antigens induce secretion of anti-inflammatory cytokines that mediate protective repair mechanisms (172). The damaging action of the toxins can be inhibited by SCFAs. Butyrate has been shown to stabilize the transcription factor HIF-1 which protects against colitis, to increase expression of epithelial tight junctions and prevent bacterial translocation (163). Also, anti-microbial peptides such as cathelicidin, NVB-302, surotomycin have been shown to be protective against *C. difficile*-mediated colitis by decreasing expression of pro-inflammatory cytokines, inhibiting toxin production, and facilitating bacterial killing of antibiotics (173–175).

The adaptive immune response does not play a direct role in the protection against CDI and disease (176). However, studies have demonstrated that an antibody-mediated immune response is important for limiting pathogenesis during the initial *C. difficile* infection, and in recurrent disease in patients. Animal models of CDI have been used to explore ways to induce protective immune responses through both passive immunization with toxin-specific antibodies and active immunization with *C. difficile* toxoids (177–180). Mucosal IgA responses may contribute to protection against CDI and associated disease, but it is dispensable. An initial CDI induces antigen-specific, mostly anti-toxin, IgA responses in patients and in animal models but the responses do not appear to correlate with protection against disease (181–185). High levels of fecal IgA are associated with protection against *C. difficile* colonization, whereas infection has been shown to significantly reduce mucosal IgA producing cells in patients (186, 187). In contrast, systemic toxin–specific IgG appears to be a better determinant of clinical outcome. IgG has been shown to protect against CDAD in both patients and animal models by decreasing gastrointestinal symptoms and mortality (188–190). *C. difficile* flagellar proteins (FliC and FliD), the surface layer proteins (SLPs), and the adhesins (Cwp66 and Cwp84) have also been shown to induce antibody responses in some patients. However, whether they play a role in the defense against recurrent disease has not been examined (191, 192).

Although the importance of antibody responses against CDI and disease has been well documented through assessment of antibody titers in patients, the cellular mechanisms required for protective B and T cell responses are not well defined. CDI is characterized by a poor initial T-cell response which can also be a marker for recurrence in CDI patients (193). Our group has recently shown that *C. difficile* infection in mice poorly activate CD4 T cells and induces a limited Tfh cell response (183).

Studies have shown that toxin A and B specific memory B cells may significantly contribute to protection against *C. difficile* associated disease. However, the high frequency of disease recurrence in *C. difficile* patients suggest that infection may not always induce a good memory response. Immunization with the inactive toxin induces toxin neutralizing antibodies and development of toxin-specific memory B cells, which confers protection against disease associated with CDI (194, 195, 197). Yet, primary *C. difficile* infection in mice induces a poor expansion of the memory B cell compartment (183). In CDI patients, anti-toxin memory B cells are detected but at a much lower frequency than in asymptomatic carriers (196).

## Conclusions and Future Directions

Humoral immune responses make a critical but incompletely understood contribution to protection against bacterial enteric pathogens. Long-lived humoral immunity, required for sustained protection relies on the orchestrated activation of professional antigen-presenting cells, T helper and T follicular helper cells, and B cells capable of differentiation into long-lived plasma cells and memory B cells. Secretion of high affinity antibodies from long-lived plasma cells and those newly differentiated from memory B cells are essential for complement-mediated bacterial clearance, and for neutralization of bacterial toxins and other virulence factors (Figure 2). As we have discussed in this article and summarized in Table 1, there are many differences in the humoral immune responses to different bacterial enteric pathogens.

**Table 1:** Overview of humoral immune responses to enteric pathogens.

	***C. rodentium***	***V. cholerae***	***Salmonella***	***Shigella***	***C. difficile***
T helper cell	• Protective IL-4+ and IL-21+ Tfh cells (41) • IL-17+, IL-22+ and IFN γ+ Th1 and Th17 cells (41, 43–46)	• IL-13+ Th2-mediated response (65) • Induction of Tfh cells (66)	• Interferon gamma-secreting T follicular cells (110)	• Protective Th17-mediated primary and recall response (150)	• Poor initial T-cell and Tfh response in patients and infected mice (183, 193)
Antibodies (Abs) and Plasma cells (PC)	• Strong Ag-specific IgA and systemic IgM and IgG (34, 35)	• Anti- toxin and LPS IgG and sIgA Abs (67–69) • Short-lived PC response (70, 71)	• Rapid Low affinity extra-follicular IgM (108) • Short-lived PC response (109)	• Serotype and antigen specific IgA, IgM and IgG (142, 143) • Slow and short-lived response especially in young patients (146, 151, 152)	• Anti-toxin IgA in mice and humans (181–185) • Poor anti-bacteria and toxin IgG (183) • Some IgM and IgG to surface proteins (191, 192)
Memory B cells (Bmem)	• Rapid germinal center in mLN (42) No study on Bmem	• Cholera-specific Bmem and rapid recall responses (66, 72–74)	• Delayed germinal center reaction (108) • IgM+ Bmem (110)	• No Bmem cells for 1st infection • LPS-specific IgA Bmem cells after 2nd challenge (145–148)	• Limited Bmem response (183, 197)
Correlates of protection against disease	• Systemic IgG translocated to gut via FcRn (36, 38–41)	• LPS and OSP-specific IgG (20, 21)	• B- cell induced IFNy+ Th1 cells and CD8 T cells, independent of secreted antibodies (70–72)	• Systemic LPS-specific IgG (144)	•Systemic anti-toxin IgG (188–190)

*C. rodentium*, a murine model for the attaching and effacing bacteria EPEC and EHEC, has been shown to induce a long-lasting immunity humoral immune response that is not always observed with other enteric pathogens. The plasma cell responses in *V. cholerae* and *Salmonella* infection are short-lived. The humoral response following *C. difficile* infection appears to be severely limited. Tfh cells and germinal center responses regulate humoral immune responses and are necessary for the induction of protective isotype-switched antibody as with *C. rodentium*. However, enteric pathogens such as *C. difficile* and *Salmonella* do not appear to induce a robust response. This limits not only the plasma cell response but also the Bmem compartment. Also, both pathogens mainly induce low affinity IgM responses.

Infections with *C. rodentium, C. difficile, V. cholerae*, and *Shigella*, promptly activate mucosal T and B cell responses leading to the production of IgA antibodies. Yet, secreted IgA appears to be dispensable for protection and bacteria-specific systemic IgG appears to be the best correlate of protection. This suggests that enteric pathogens or their antigens need to reach the extra-intestinal lymphoid organs to induce a systemic antibody response. Also, it suggests that there must be mechanisms by which IgG is translocated to the intestinal lumen to limit disease and bacterial dissemination. The neonatal Fc receptor (FcRn) mediates this IgG transport in *C. rodentium* (31), but little is known about the mechanism in the other pathogens.

As regards humoral immunity to enteric bacterial pathogens, future directions may include:

- More complete investigation of the Th/Tfh, Bmem cell, and plasma cell responses to infection in both animal models and human patients.
- Determining the mechanisms of antigen presentation in the gut, and how this influences activation and programming of B cell and Th/Tfh responses.
- Determining whether secreted toxins subvert humoral immunity and the mechanisms by which this is achieved.
- A systematic genomic approach to obtain in-depth immune profiles of the humoral response to infection and immunization.
- Single cell BCR sequencing to determine the plasma cell and memory B cell repertoires.

The application of newer technologies to the humoral response to enteric pathogens may be particularly important for infections with *C. difficile* and *Salmonella* that are characterized by persistent and/or recurrent disease. This may lead to a better understanding of the mechanisms by which memory B cell and plasma cell responses are limited, and could contribute to determining causes of persistent or recurrent disease.

## Author Contributions

SA and ML wrote and edited the article. SA devised and produced the illustrations. All authors contributed to the article and approved the submitted version.

## Conflict of Interest

The authors declare that the research was conducted in the absence of any commercial or financial relationships that could be construed as a potential conflict of interest.
